# Supplementary material for: Tea Tree Oil Mediates Antioxidant Factors Relish and Nrf2-Autophagy Axis Regulating the Lipid Metabolism of Macrobrachium rosenbergii
Source: Antioxidants (Basel). 2022 Nov 16;11(11):2260. doi: 10.3390/antiox11112260 (PMC9686997; doi:10.3390/antiox11112260)
Supplement: Supplementary file 1 [file antioxidants-11-02260-s001.zip › antioxidants-1974174-supplementary.pdf]

**Table S1:** Primers used in the study.

| Gene | Primer | Sequences (5′-3′) | Sequence source |
|---|---|---|---|
| *β-actin* | F | TCCGTAAGGACCTGTATGCC | Tadese et al. 2021 |
| | R | TCGGGAGGTGCGATGATTTT | AY651918.2 |
| *6pgd* | F | GACTTCCGCACTAACCCACT | |
| | R | AGCTTCAGGACTGTTCCACC | |
| *Acca* | F | TCATTGGGAGCAACATCA | |
| | R | GCGATTAGGACAGCGAGT | |
| *Fasn* | F | TGGTGATCTTCCTCCAGCGT | |
| | R | ACGTAGGACACATCTGAGGGA | |
| *Dgat* | F | TCACAACCCGACAGACCG | |
| | R | TGGCGTTTGGAAATAGTAGC | |
| *G6pd* | F | GACGATCAAGCGACACGA | |
| | R | CAAGGTCACCCGAAGCAC | |
| *Atgl* | F | ATGGAGGGAGAAGTGAGT | |
| | R | ATAGACGGAGGTATGAGC | |
| *Cpt1* | F | TGATGGATGATGAAGCCTAT | |
| | R | TCCTCCCACCAATCTGTT | |
| *Hsl* | F | GCAGATATGCCGCTAACA | |
| | R | TCCCGAACAGATAAACAGG | |
| *Ampkα* | F | TGAAAGGGCGCTAAGTGGAG | |
| | R | ACTCGCACATGGAAAGGGTT | |
| *Srebp1* | F | AGGGCTATTGTGAGATGA | |
| | R | CTGTAGATGTTTATTGGGTT | |
| *Fatp1* | F | TTTATGACTGCTGGTGTAT | |
| | R | TGTAGAACAGCCAAAGAG | |
| *Fabp7* | F | TAGACTCGCTTGCTGACA | |
| | R | TACAATTACCCTGGATGG | |
| *Pparα* | F | ATGTCCAGTCCTCCTGTACTCC | |
| | R | AAGAGCAGAGCCGCAATCAG | |
| *Pparγ* | F | TCTGTTACCGCTGGTGGCTG | |
| | R | TGTGAACCGTTTAGTGTTTTGGC | |
| *Keap1* | F | TTCCACGCCCTCCTCAA | KF811013 |
| | R | TGTACCCTCCCGCTATG | |
| *Nrf2* | F | CTGGACGAGGAGACTGGA | KF733814 |
| | R | ATCTGTGGTAGGTGGAAC | |
| *Imd* | F | CGACCACATTCTCCTCCTCCC | |
| | R | TTCAGTGCATCCACGTCCCTC | |
| *Relish* | F | GATGAGCCTTCAGTGCCAGA | KR827675.1 |
| | R | CCAGGTGACGCCATGTATCA | |

| | | |
|---|---|---|
| *Ampk* | F | TTACTGCCACCGTCACATGG |
| | R | TGGGGCAGCATAGTTTGGAG |
| *Akt* | F | GGTCCAAACATGCTCAACCAA |
| | R | AGGTGTTCCCTACTTTCACTTGC |
| *Mtor* | F | GAGACTTCTGCCACGACGAA |
| | R | CAAAACGGGCACCAACCAAT |
| *Ulk1* | F | CATAAGCCGACCATCTCCCC |
| | R | CACATGGGAGGAGTGCCAAT |
| *Beclin 1* | F | CAGCTTCATTCGGGAGTGGT |
| | R | ACGGAGAACGAAAGGTGTCC |
| *Atg13* | F | GTATAAAGTGCGTTCGCCCC |
| | R | AATGACGTGGTCGTGGAAGT |
| *Atg3* | F | TTGGACAATTCCAGTCCCACA |
| | R | CCCTACTTTCACTTGCATCCTTG |
| *Atg4b* | F | AGGCATCCGTTGAGCAAAAG |
| | R | ACAAGGGATGGGGCTGTATG |
| *Atg5* | F | GCAAAGCTGTTCCACCACAA |
| | R | TGATGGTTCCAAGGCTGTCG |
| *Atg7* | F | CGAGTAGGAAGCCCTTGTGG |
| | R | TGTTGATGCAGGCTTTTGGC |
| *Lc3* | F | AGCGTATTCACCTTCGACCA |
| | R | AAGTCCTCTTCGTGGTGTTCC |
| *P62* | F | ATCAACAGAGTCCGCGACAA |
| | R | GGCACCAACCAATGTAGCAC |
